# Evaluation of liver function in patients with liver cirrhosis and chronic liver disease using functional liver imaging scores at different acquisition time points

**DOI:** 10.3389/fgene.2022.1071025

**Published:** 2022-12-06

**Authors:** Guixiang Tang, Jianbin Liu, Peng Liu, Feng Huang, Xunuo Shao, Yao Chen, An Xie

**Affiliations:** ^1^ Department of Radiology, Hunan Provincial People’s Hospital (The First Affiliated Hospital of Hunan Normal University), Changsha, China; ^2^ School of Mathematics and Statistics, Hunan Normal University, Changsha, China

**Keywords:** functional liver imaging scores, magnetic resonance imaging, chronic liver disease, liver function evaluation, Gd-EOB-DTPA

## Abstract

**Purpose:** This paper aims to explore whether functional liver imaging score (FLIS) based on Gd-EOB-DTPA-enhanced magnetic resonance imaging (MRI) images at 5, 10, and 15 min can predict liver function in patients with liver cirrhosis or chronic liver disease and its association with indocyanine green 15-min retention rate (ICG-R_15_), Child-Pugh (CP) score, albumin-bilirubin (ALBI) score, and model for end-stage liver disease (MELD) score. In addition, it also examines the inter- and intra-observer consistency of FLIS and three FLIS parameters at three different time points.

**Methods:** This study included 110 patients with chronic liver disease (CLD) or liver cirrhosis (LC) (93 men, 17 women; mean ± standard deviation = 56.96 ± 10.16) between July 2019 and May 2022. FLIS was assigned in accordance with the sum of the three hepatobiliary phase characteristics, all of which were scored on the 0–2 ordinal scale, including the biliary excretion, hepatic enhancement and portal vein signal intensity. FLIS was calculated independently by two radiologists using transitional and hepatobiliary phase images at 5, 10, and 15 min after enhancement. The relationship between FLIS and three FLIS quality scores and the degree of liver function were evaluated using Spearman’s rank correlation coefficient. The ability of FLIS to predict hepatic function was investigated using receiver operating characteristic (ROC) curves.

**Results:** Intra- and inter-observer intraclass correlation coefficients (ICCs) (ICC = 0.937–0.978, 95% CI = 0.909–0.985) for FLIS at each time point indicated excellent agreement. At each time point, FLIS had a moderate negative association with liver function classification (*r* = [−0.641]-[−0.428], *p* < 0.001), and weak to moderate correlation with some other clinical parameters except for creatinine (*p* > 0.05). FLIS showed moderate discriminatory ability between different liver function levels. The area under the ROC curves (AUCs) of FLIS at 5, 10, and 15 min after enhancement to predict ICG-R_15_ of 10% or less were 0.838, 0.802, and 0.723, respectively; those for predicting ICG-R_15_ greater than 20% were 0.793, 0.824, and 0.756, respectively; those for predicting ICG-R_15_ greater than 40% were 0.728, 0.755, and 0.741, respectively; those for predicting ALBI grade 1 were 0.734, 0.761, and 0.691, respectively; those for predicting CP class A cirrhosis were 0.806, 0.821, and 0.829, respectively; those for predicting MELD score of 10 or less were 0.837, 0.877, and 0.837, respectively. No significant difference was found in the AUC of FLIS at 5, 10 and 15 min (*p* > 0.05).

**Conclusion:** FLIS presented a moderate negative correlation with the classification system of hepatic function at a delay of 5, 10, and 15 min, and patients with LC or CLD were appropriately stratified based on ICG-R_15_, ALBI grade, MELD score, and CP classification. In addition, the use of FLIS to evaluate liver function can reduce the observation time of the hepatobiliary period.

## 1 Introduction

Liver function estimation is a key clinical issue in monitoring the development of chronic liver disease (CLD), determining optimal treatment strategies, and preventing liver failure after surgical treatment (Yamada et al., 2011; Ba-Ssalamah A et al., 2017). In clinical practice, liver reserve function is often evaluated by Child-Pugh (CP) score, indocyanine green 15-min retention rate (ICG-R_15_), albumin-bilirubin (ALBI) score, and model for end-stage liver disease (MELD) score (Yamada et al., 2011; Hiraoka et al., 2017; Wang et al., 2018; Wang et al., 2020; Demirtas et al., 2021).

Gadolinium-ethoxy benzyl-diethylene triamine pentaacetic acid (Gd-EOB-DTPA) is a paramagnetic hepatobiliary-specific magnetic resonance imaging (MRI) contrast agent that is easily absorbed and secreted into the biliary system by organic anion transport polypeptides (OATPs) on normal hepatocytes during the transition period (3–5 min after contrast agent injection) and hepatobiliary phase (HBP) (20 min) but without any change in its chemical structure. Moreover, Gd-EOB-DTPA is featured by both non-specific extracellular contrast agents and hepatocyte-specific contrast agents (Hamm et al., 1995; Reimer et al., 2004; Van Beers et al., 2012). Recently, some studies have shown that various quantitative parameters based on Gd-EOB-DTPA-enhanced MRI HBP images, including relative liver enhancement, contrast enhancement index, hepatocyte uptake index and T1 value, have a high correlation with the clinical liver function assessment system. It has also been demonstrated that these parameters can be used as non-invasive imaging tools to evaluate the level of liver function (Wibmer et al., 2013; Lee et al., 2016; Yoon et al., 2017; Yoon et al., 2019; Li et al., 2021). However, it is well-known that these methods have limitations, such as complex computation and modeling, dependence on vendor, field strength and sequence, and poor repeatability.

Recently, Bastati et al. (2016) proposed a simple, semi-quantitative FLIS based on the three HBP visual characteristics of Gd-EOB-DTPA-enhanced MRI, including liver parenchymal enhancement quality score (EnQS), biliary contrast excretion quality score (ExQS), and portal vein sign quality score (PVsQS), and each score was given 0–2 points according to the criteria. Since FLIS requires no signal intensity measurement, equation calculation or specific software, and is independent of MRI field-strength and vendor, it is easier to perform in routine clinical practice than other quantitative parameters-based Gd-EOB-DTPA-enhanced MRI. According to Bastati et al., FLIS can predict the survival rate of transplanted livers within patients after liver transplantation and represents the independent risk factor for the first hepatic decompensation and death within patients that suffer from advanced CLD (Bastati et al., 2016; Bastati et al., 2020). Similarly, an algorithm based on splenic craniocaudal diameter and FLIS has also been reported to independently predict transplant-free mortality and to effectively stratify transplant-free survival in patients with advanced CLD (Bastati et al., 2022). Furthermore, FLIS is strongly correlated with CP score and ALBI score in patients with liver cirrhosis (LC) and CLD, and patients can be classified according to CP grade or ALBI grade (Lee et al., 2021; Aslan et al., 2022). However, it was reported by Luo et al. (2022) that FLIS was weakly linked to ICG-R_15_, ALBI score, and MELD score. In addition, studies also pointed out that FLIS and most liver resection were independent predictors of PHLF, whose predictive ability is likely to be better than ICG-R_15_, ALBI score and MELD score. It has been shown that about 20 min after Gd-EOB-DTPA injection, hepatocytes reach a plateau and last for about 2 h. Thus, the pharmacokinetics of Gd-EOB-DTPA has a slow speed, leading to the failure of observing a complete process of excretion and accumulation of contrast agents over time in a standard clinical setting (Ba-Ssalamah et al., 2009). In clinical practice, a time lapse of 20 min is often used to collect images of the hepatobiliary stage. The uptake of Gd-EOB-DTPA in hepatocytes is responsible for hepatobiliary signal changes during the hepatobiliary stage. However, the correlation between FLIS and ICG-R_15_, ALBI score, CP score and MELD score, as well as the change of FLIS with different scanning times in patients with CLD and LC, at different acquisition time points has remained unclear.

Therefore, this study aims to explore whether FLIS based on Gd-EOB-DTPA-enhanced MRI images at 5, 10, and 15 min can predict liver function in patients with LC or CLD, and its association with ICG-R_15_, CP score, ALBI score, and MELD score. Furthermore, it also examines the inter- and intra-observer consistency of FLIS and three FLIS parameters at three different time points.

## 2 Materials and methods

### 2.1 Study subject

**FIGURE 1:**
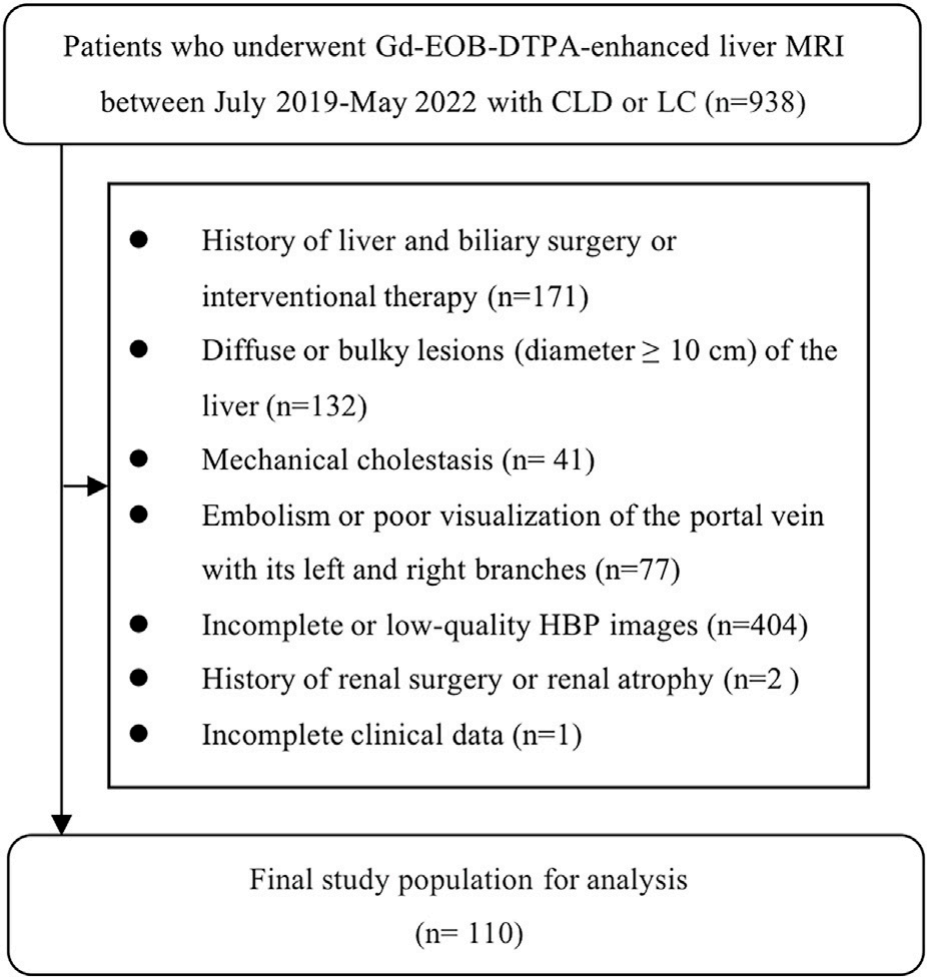
The results of the patients' enrolments. In total, 110 patients were enrolled in this study.

938 patients from Hunan Provincial People’s Hospital were enrolled from July 2019 to May 2022 according to the inclusion criteria (Figure 1).

The inclusion criteria were as follows: 1) Gd-EOB-DTPA-enhanced MRI scanning; 2) clinical or histologic evidence of LC or CLD; 3) the interval between routine laboratory examination and ICG clearance test and MRI examination was less than 2 weeks.

The exclusion criteria were as follows: 1) history of liver and biliary surgery or interventional therapy; 2) diffuse or bulky lesion (diameter ≥10 cm) of the liver; 3) mechanical cholestasis; 4) embolism or poor visualization of the portal vein with its left and right branches; 5) incomplete or low-quality HBP images; 6) history of renal surgery or renal atrophy; 7) incomplete clinical data.

Consequently, this study included 110 patients, containing 93 men (85%) and 17 women (15%), at the age of 31–86 (mean ± SD = 56.96 ± 10.16). Thereinto, there were a total of 78 patients complicated with HCC, 2 complicated with ICC, and 3 complicated with hepatic metastases from intestinal malignancies. The work was agreed upon by the Ethics Committee of the Hunan Provincial People’s Hospital (The First Affiliated Hospital of Hunan Normal University). Due to the retrospective nature of this study, the informed consent was waived.

### 2.2 Clinical data

A diagnostic radiologist recorded and reviewed clinical and demographic data of patients, including gender, age, clinical information (potential liver disease, hepatic encephalopathy, ascites), serum markers [bilirubin, albumin, creatinine, prothrombin time (PT), international normalized ratio (INR)] and ICG-R_15_. In addition, ALBI score, CP score, and MELD score were obtained, among which MELD score = 11.29 ln [INR] + 3.789 ln [bilirubin (umol/L)/17.1] + 9.579 ln [creatinine (umol/L)/88.4] + 6.43. Liver disease severity was classified based on the CP score, ALBI score and MELD score, which is an internationally recognized scoring system for the evaluation of CLD severity. If the MELD score is equal to or less than 10, it represents a healthy condition; if the MELD score ranges from 11 to 18, it represents moderate liver disease; if the MELD score is greater than 18, it represents severe liver disease (Wiesner et al., 2003).

### 2.3 Magnetic resonance imaging

All patients underwent MR examination using 3.0-T scanners (Ingenia, Philips Healthcare, Best, the Netherlands) with a 32-channel phased array body coil as the receiving coil. MRI was used to scan the traditional cross-section T2WI with spectral attenuated inversion recovery (SPAIR) sequence, chemical shift imaging (positive and negative phases T1WI) and DWI (*b* values of 0, 400, and 800 s/mm^2^). Gd-EOB-DPTA (Primovist; Bayer Schering Pharma AG, Berlin, Germany) was administrated by peripheral intravenous injection at a rate of 1.0 mL/s as an enhanced scanning contrast agent at a dose of 0.1 mL/kg body weight, followed by a 20 mL saline flush at once. Images were gathered from equilibrium, portal venous, and arterial phases at 20, 60, and 90 s after enhancement. The whole liver was scanned through the use of the scanning of the liver enhanced T1 high-resolution isotropic volume excitation (THRIVE).

Transitional phase and HBP images were gathered at 5, 10, and 15 min after Gd-EOB-DTPA injection. The parameters included TE = 2.3 ms, TR = 3.6 ms, section thickness = 4.0 mm, flip angle = 15°, slice gap = −2.0 mm, NSA = 1, FOV = 420 × 315 mm^2^, matrix = 232 × 174. In the process of HBP imaging, a 20-min delay was not observed as a 15-min delay is sufficient to capture HBP (Liang et al., 2016). The technical parameters of MRI sequences are detailed in Table 1.

**TABLE 1:** Technical details of MRI scanning parameters. The DWI sequences involved the ADC map calculation. T2-WI, T2-weighted images; DWI, diffusion-weighted images; HBP, hepatobiliary phase; T1-WI, T1-weighted images; DCE, dynamic contrast enhancement; SPIR, spectral presaturation with inversion recovery; TSE, turbo spin echo; SPAIR, spectral attenuated inversion recovery; FFE, fast field echo; EPI, echo planar imaging; TE, time to echo; TR, time to repetition; FOV, field of view.

Parameter	T2-WI	T1-WI in-phase	T1-WI opposed-phase	DWI	DCE	HBP
Imaging planes	Axial-coronal	Axial	Axial	Axial	Axial-coronal	Axial-coronal
Fat saturation	SPAIR	—	—	SPIR	SPAIR	SPAIR
Sequence	TSE	FFE	FFE	EPI	FFE	FFE
TR (ms)	2725	3.6	3.6	2000	3.6	3.6
TE (ms)	76	1.32	2.3	67	2.3	2.3
Flip Angle (degree)	90	15	15	90	15	15
FOV (mm^2^)	360 × 360	420 × 315	420 × 315	400 × 376	420 × 315	420 × 315
Slice thickness	5	4	4	4	4	4
Slice gap	0.5	−2	−2	0.4	−2	−2
Matrix size	240 × 240	232 × 174	232 × 174	132 × 122	232 × 174	232 × 174
*b* value (s/mm^2^)	—	—	—	0, 400, 800	—	—
Number of acquisitions	—	—	—	—	5	3

### 2.4 Image analysis

**FIGURE 2:**
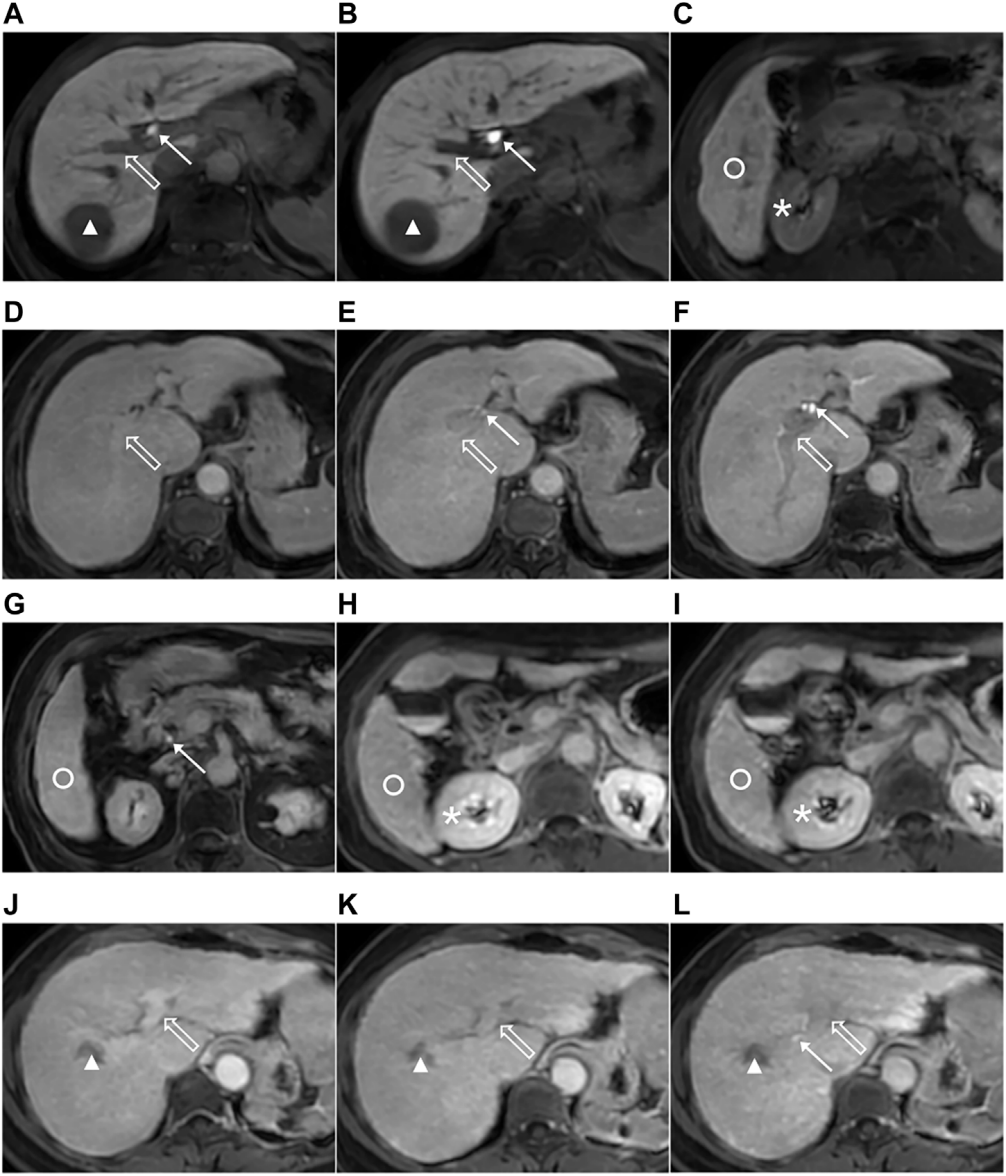
Images from 3.0-T MRI that were obtained 5, 10, and 15 min after gadoxetic acid was administrated within axial planes in the three patients that suffer from the chronic liver disease or the liver cirrhosis. The 51-year-old man that suffer from the chronic hepatitis B, hepatocellular carcinoma (HCC) (triangle), ICG-R_15_ of 3.6%, ALBI grade 1, CP A cirrhosis and MELD score of 8 with the functional liver imaging score (FLIS) of 6 at 5 **(A,C)**, 10 **(B)** and 15 min (not shown). Liver parenchymal enhancement quality score (EnQS) is 2 at each time point due to that the signal intensity of the liver (circle) is higher compared to that of the right kidney (*). Excretion quality score (ExQS) is 2 at each time point due to that the contrast media is found to be within the common bile duct (solid arrow). Portal vein sign quality score (PVsQS) is 2 at each time point due to that the portal vein (hollow arrow) is seen to be hypointensed to the liver parenchyma (circle). A 78-year-old woman with cryptogenic liver disease, HCC (not shown), ICG-R_15_ of 6.8%, ALBI grade 2, CP A cirrhosis and MELD score of 15 with functional liver imaging score (FLIS) of 2, 3 and 5 at 5 **(D)**, 10 **(E)** and 15 min **(F,G)**, respectively. EnQS is 1 at each time point due to that there is equal signal intensity of the liver (circle) and the right kidney (*). ExQS is 0, 1, and 2 at 5, 10, and 15 min, respectively, due to that contrast media is not presented, excreted into the intrahepatic bile ducts and common bile duct respectively (solid arrow). PVsQS is 1 at 5 and 10 min and 2 at 15 min due to that the portal vein (hollow arrow) is found to be both isointense and hypointense to the liver parenchyma (circle), respectively. A 59-year-old woman that suffers from the hepatitis C virus cirrhosis, HCC (triangle), ICG-R_15_ of 21.0%, ALBI grade 2, CP A cirrhosis and MELD score of 7 with the functional liver imaging score (FLIS) being 0, 1 and 3 at 5 **(J)**, 10 **(H,K)** and 15 min **(I, L)**, respectively. EnQS is 0 at 5 and 10 min and 1 at 15 min due to that the signal intensity of the liver (circle) is both lower than and equals to that of the right kidney (*), respectively. ExQS is 0 at 5 and 10 min and 1 at 15 min because contrast media is not shown and excreted into the intrahepatic bile ducts (solid arrow). PVsQS is 0 at 5 min and 1 at 10 and 15 min due to that the portal vein (hollow arrow) is both hyperintens and isointense to the liver parenchyma (circle), respectively.

Two radiologists, with 2-year abdominal imaging experience and training before the evaluation, independently analyzed the transitional and hepatobiliary phase-enhanced MRI image at 5, 10, and 15 min after the Gd-EOB-DTPA was administered on the picture archiving and communication system (PACS). Radiologist 1 analyzed all MRI images again to evaluate intra-observer repeatability after a 4-week interval. The radiologists were blinded to the clinical and medical information of patients. Following the grading system for FLIS parameters, the three quality scores, which were the liver parenchymal enhancement (EnQS), the biliary contrast excretion (ExQS) and the portal vein sign (PVsQS) were all decided (Bastati et al., 2016) (Table 2). The score for each parameter ranged from 0 to 2. FLIS indicates the sum of the three quality scores, ranging from 0 to 6 (Figure 2).

**TABLE 2:** Defnition and grading system for the three FLIS parameters.

Parameter	Points
Liver parenchymal enhancement quality score	
SI of liver parenchyma relative to kidney on HBP	
Hypointense	0
Isointense	1
Hyperintense	2
Biliary contrast excretion quality score	
Presence of contrast media in the bile ducts on HBP	
No biliary contrast excretion	0
Excretion into peripheral intrahepatic bile ducts or the right and/or left hepatic duct(s)	1
Excretion into the common hepatic duct, the common bile duct, or the duodenum	2
Portal vein sign PVsQS	
SI of the portal vein relative to the liver parenchyma on HBP	
Hyperintens	0
Isointense	1
Hypointense	2

SI, signal intensity; HBP, hepatobiliary phase; PVsQS, portal vein sign quality score.

### 2.5 Statistical analysis

R software (version 4.2.0) and IBM SPSS statistical software (version 26.0) were used for all statistical analyses. The Kolmogorov-Smirnov test was used to evaluate the normality of data. For normally distributed data, mean and standard deviation (SD) was used to report continuous variables, and median with interquartile ranges (IQR) was for skewed data, while the number and percentage of patients with specific features were used to report categorical variables. The mixed intraclass correlation coefficient (ICC) model was employed to obtain inter- and intra-observer agreement, with a absolute agreements, single measurements, and 95% confidence intervals (CIs). Poor reliability was indicated as a value of less than 0.5, moderate reliability was indicated as a value of 0.5–0.75, good reliability was indicated as a value of 0.75–0.9, and excellent reliability was indicated as a value of greater than 0.9 (Koo and Li, 2016). The relationship between FLIS and each of the three FLIS quality scores and the degree of liver function, was assessed using Spearman’s rank correlation coefficient (*r*). The receiver operating characteristic (ROC) curve analysis was used to estimate the discriminatory ability of variables, and the Youden index (sensitivity + specifificity-1) was used to estimate the optimal cut-off values (Akobeng A.K., 2007). The area under the ROC curves (AUCs) were compared using the Delong test. A statistically significant difference was expressed as the two-sided *p*-value of less than 0.05.

## 3 Results

Images were collected at 5, 10, and 15 min after enhancement of Gd-EOB-DTPA. The final three FLIS quality scores and FLIS were obtained at each time point based on the image information.

### 3.1 Baseline information and feature screening

Table 3 shows the clinical characteristics of patients. This study included 110 patients, containing 93 men and 17 women, at the age of 31–86 (mean ± SD = 56.96 ± 10.16). The etiologies of LC and CLD were as follows: hepatitis B virus (91 of 110 patients, 82.7%), hepatitis C virus (9 of 110 patients, 8.2%), alcoholic liver disease (4 of 110 patients, 3.6%), non-alcoholic fatty liver (2 of 110 patients, 1.8%), cryptogenic liver disease (2 of 110 patients, 1.8%), coinfection with hepatitis B and C (1 of 110 patients, 0.9%), and autoimmune hepatitis (1 of 110 patients, 0.9%). Thereinto, there were a total of 78 patients complicated with HCC, 2 complicated with ICC, and 3 complicated with hepatic metastases from intestinal malignancies. Among the main causes of CLD or LC in the HCC cohort, 87.2% (68 of 78 patients) were hepatitis B virus, and 7.7% (6 of 78 patients) were hepatitis C virus. The ALBI score indicated that all of the patients were classified into ALBI grade 1 (42 of 110 patients, 38.2%), ALBI grade 2 (60 of 110 patients, 54.5%), and ALBI grade 3 (8 of 110 patients, 7.3%) respectively. According to the CP score, patients were divided into CP A cirrhosis (80 of 110 patients, 72.7%), CP B cirrhosis (25 of 110 patients, 22.7%), and CP C cirrhosis (5 of 110 patients, 4.5%).

**TABLE 3:** Baseline characters of the study population. HCC, hepatocellular carcinoma; ICC, intrahepatic cholangiocarcinoma.

Characteristics	All patient (n = 110)
Gender	
Male	93 (85%)
Female	17 (15%)
Age (years)	
Mean ± SD	56.96 ± 10.16
Range	31–86
Etiology of liver disease	
Hepatitis B virus	91 (82.7%)
Hepatitis C virus	9 (8.2%)
Alcoholic liver disease	4 (3.6%)
Non-alcoholic fatty liver	2 (1.8%)
Cryptogenic liver disease	2 (1.8%)
Coinfection with hepatitis B and C	1 (0.9%)
Autoimmune hepatitis	1 (0.9%)
Complicated with HCC	78 (70.9%)
Complicated with ICC	2 (1.8%)
Hepatic metastases (intestinal origin)	3 (2.7%)
Serum markers	
Albumin (g/L)	37.46 ± 6.56
Total bilirubin (umol/L)	18.06 (12.25–27.65)
Creatinine (umol/L)	68.25 (59.00–76.36)
PT (s)	11.80 (10.80–12.98)
INR	1.03 (0.94–1.15)
ICG-R_15_	11.45 (5.43–28.40)
≤10%	49 (44.5%)
>20%	37 (33.6%)
>40%	14 (12.7%)
ALBI grade	−2.35 ± 0.65
1	42 (38.2%)
2	60 (54.5%)
3	8 (7.3%)
CP class	5 (5–7)
A	80 (72.7%)
B	25 (22.7%)
C	5 (4.5%)
MELD score	7 (4–10)
≤10	87 (79.1%)
10–18	22 (20.0%)
>18	1 (0.9%)

### 3.2 Intra- and inter-observer agreement for three FLIS parameters and FLIS

Intra-observer ICCs for FLIS at 5, 10, and 15 min after enhancement were 0.96 (95% CI: 0.95, 0.98), 0.98 (95% CI: 0.97, 0.99) and 0.98 (95% CI: 0.97, 0.99), respectively. Inter-observer ICCs for FLIS at 5, 10, and 15 min after enhancement were 0.94 (95% CI: 0.91, 0.96), 0.94 (95% CI: 0.91, 0.96) and 0.95 (95% CI: 0.93, 0.97), respectively. Intra- and inter-observer ICCs for FLIS at each time point indicated excellent agreement. Furthermore, the range of ICCs for the three quality scores was as follows: for the intra-observer agreement, 0.92–0.95 (95% CI: 0.88, 0.97), 0.95–1.00 (95% CI: 0.93, 1.00) and 0.95–0.97 (95% CI: 0.92, 0.98); for the inter-observer agreement, 0.84–0.90 (95% CI: 0.74, 0.93), 0.91–0.93 (95% CI: 0.87, 0.95) and 0.84–0.90 (95% CI: 0.77, 0.93); and EnQS, ExQS, and PVsQS, respectively (Table 4). Thus, the level of intra- and inter-observer reliability of the three FLIS parameters was “good” to “excellent” and “moderate” to “good,” respectively (Koo and Li, 2016). The subsequent results are provided only for radiologist 1.

**TABLE 4:** Intra- and inter-observer agreement for FLIS and the three FLIS parameters at different time points.

Parameters	Interobserver ICCs (95% CI)			Intraobserver ICCs (95% CI)		
	5 min	10 min	15 min	5 min	10 min	15 min
FLIS	0.940 (0.913, 0.958)	0.937 (0.909, 0.957)	0.952 (0.929, 0.968)	0.964 (0.947, 0.975)	0.978 (0.969, 0.985)	0.978 (0.968, 0.985)
EnQS	0.876 (0.823, 0.914)	0.844 (0.741, 0.902)	0.899 (0.847, 0.933)	0.916 (0.880, 0.942)	0.921 (0.887, 0.945)	0.953 (0.932, 0.968)
ExQS	0.908 (0.869, 0.936)	0.925 (0.891, 0.948)	0.923 (0.889, 0.946)	0.954 (0.933, 0.968)	0.988 (0.983, 0.992)	1.000 (1.000, 1.000)
PVsQS	0.895 (0.851, 0.927)	0.838 (0.773, 0.886)	0.841 (0.777, 0.888)	0.945 (0.921, 0.962)	0.945 (0.921, 0.962)	0.968 (0.953, 0.978)

### 3.3 Correlation of FLIS and three FLIS parameters with ICG-R_15_, ALBI score, CP score, MELD score and other clinical parameters

**FIGURE 3:**
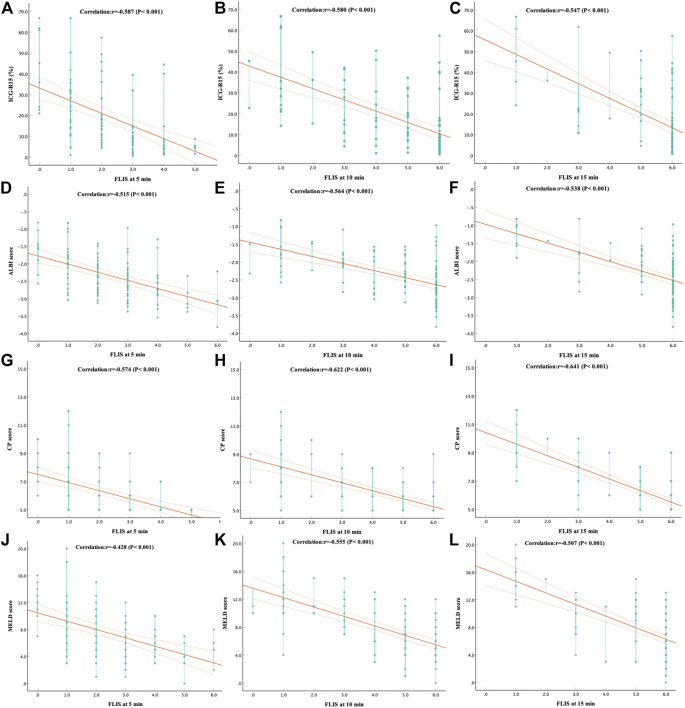
The correlation between **(A–C)** ICG-R_15_, **(D–F)** ALBI score, **(G–I)** CP score and **(J–L)** MELD score and FLIS at different time points. The correlation was assessed by using the Spearman rank correlation analysis. In addiiton, FLIS, functional liver imaging score; ICG-R_15_, indocyanine green retention rate at 15 min; ALBI, albumin-bilirubin; CP, child-pugh; MELD, model for end-stage liver disease.

Spearman’s rank correlation analysis was performed in a total of 110 patients with CLD and LC for the grading system of FLIS and three FLIS parameters by referring to the ICG-R_15_, the ALBI score, the CP score, the MELD score, and some other clinical parameters, respectively (Tables 5, 6, Figure 3). FLIS had a moderate negative correlation with liver function classification (*r* = [−0.641]-[−0.428], *p* < 0.001), and was weak to moderate correlation with other clinical parameters except for creatinine (*r* = 0.018–0.128, *p* > 0.05), at per timepoint. Overall, FLIS at 10 and 15 min after enhancement showed the strongest correlation with CP score (r = −0.622, r = −0.641; *p* < 0.001). Among the three FLIS parameters, ExQS showed a lower correlation than EnQS and PVsQS at each time point. In addition, there was no connection between ExQS at 5 min after enhancement, with CP score and MELD score, between ExQS at 5- and 15-min delay and INR and PT, as well as between FLIS and its three parameters at each time point and creatinine (*p* > 0.05).

**TABLE 5:** Correlations to ICG-R_15_, ALBI score, CP score and MELD score for FLIS and three FLIS parameters at different time points.

Correlation	ICG-R_15_		ALBI score		CP score		MELD score	
	Coefficient	P	Coefficient	P	Coefficient	P	Coefficient	P
At a 5-min delay								
FLIS	−0.587	<0.001	−0.515	<0.001	−0.574	<0.001	−0.428	<0.001
EnQS	−0.559	<0.001	−0.492	<0.001	−0.536	<0.001	−0.355	<0.001
ExQS	−0.210	0.027	−0.233	0.014	−0.186	0.052	−0.164	0.086
PVsQS	−0.537	<0.001	−0.458	<0.001	−0.529	<0.001	−0.431	<0.001
At a 10-min delay								
FLIS	−0.580	<0.001	−0.564	<0.001	−0.622	<0.001	−0.555	<0.001
EnQS	−0.632	<0.001	−0.629	<0.001	−0.678	<0.001	−0.531	<0.001
ExQS	−0.361	<0.001	−0.318	0.001	−0.373	<0.001	−0.458	<0.001
PVsQS	−0.468	<0.001	−0.495	<0.001	−0.601	<0.001	−0.518	<0.001
At a 15-min delay								
FLIS	−0.547	<0.001	−0.538	<0.001	−0.641	<0.001	−0.507	<0.001
EnQS	−0.542	<0.001	−0.567	<0.001	−0.666	<0.001	−0.537	<0.001
ExQS	−0.376	<0.001	−0.284	0.003	−0.349	<0.001	−0.325	0.001
PVsQS	−0.405	<0.001	−0.455	<0.001	−0.546	<0.001	−0.369	<0.001

**TABLE 6:** Correlations to clinical parameters for FLIS at different time points.

Correlation	Albumin (g/L)		Bilirubin (umol/L)		Creatinine (umol/L)		PT (s)		INR	
	Coefficient	P	Coefficient	P	Coefficient	P	Coefficient	P	Coefficient	P
At a 5-min delay										
FLIS	0.455	<0.001	−0.448	<0.001	0.128	0.181	−0.353	<0.001	−0.354	<0.001
EnQS	0.466	<0.001	−0.386	<0.001	0.156	0.104	−0.334	<0.001	−0.334	<0.001
ExQS	0.216	0.023	−0.216	0.023	0.043	0.656	−0.096	0.319	−0.096	0.319
PVsQS	0.386	<0.001	−0.418	<0.001	0.105	0.274	−0.328	<0.001	−0.329	<0.001
At a 10-min delay										
FLIS	0.485	<0.001	−0.527	<0.001	0.018	0.850	−0.426	<0.001	−0.426	<0.001
EnQS	0.569	<0.001	−0.488	<0.001	0.032	0.739	−0.480	<0.001	−0.480	<0.001
ExQS	0.231	0.015	−0.491	<0.001	0.047	0.628	−0.261	0.006	−0.262	0.006
PVsQS	0.452	<0.001	−0.405	<0.001	0.058	0.546	−0.410	<0.001	−0.407	<0.001
At a 15-min delay										
FLIS	0.481	<0.001	−0.485	<0.001	0.105	0.276	−0.424	<0.001	−0.424	<0.001
EnQS	0.507	<0.001	−0.504	<0.001	0.098	0.310	−0.467	<0.001	−0.466	<0.001
ExQS	0.223	0.019	−0.363	<0.001	0.038	0.694	−0.169	0.077	−0.172	0.073
PVsQS	0.390	<0.001	−0.401	<0.001	0.131	0.172	−0.340	<0.001	−0.338	<0.001

### 3.4 ROC analysis of FLIS for classification of ICG-R_15_, ALBI grade, CP score and MELD score

**FIGURE 4:**
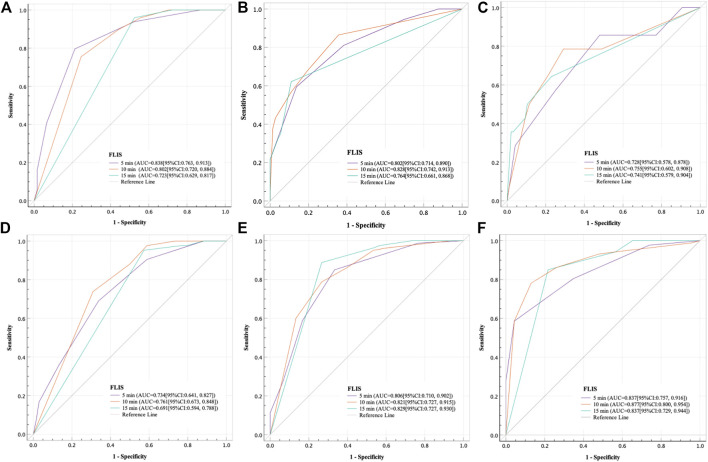
Receiver operating characteristic curve analysis of the FLIS for the purpose of **(A)** predicting ICG-R_15_ ≤ 10%, **(B)** predicting ICG-R_15_ > 20%, **(C)** predicting ICG-R_15_ > 40%, **(D)** predicting the ALBI grade 1, **(E)** predicting CP A cirrhosis and **(F)** predicting MELD score ≤10 at different time points. In addition, FLIS, functional liver imaging score; ICG-R_15_, indocyanine green retention rate at 15 min; ALBI, albumin-bilirubin; CP, child-pugh; MELD, model for end-stage liver disease.

The results of the ROC curve analysis of FLIS at 5, 10, and 15 min after enhancement are shown in Table 7. The AUCs of FLIS at 5, 10, and 15 min after enhancement for predicting ICG-R_15_ of 10% or less were 0.838 (95% CI: 0.763, 0.913, *p* < 0.001), 0.802 (95% CI: 0.720, 0.884, *p* < 0.001) and 0.723 (95% CI: 0.629, 0.817, *p* < 0.001), respectively. The AUCs of FLIS at 5, 10, and 15 min after enhancement for predicting ICG-R_15_ greater than 20% were 0.793 (95% CI: 0.703, 0.883, *p* < 0.001), 0.824 (95% CI: 0.737, 0.912, *p* < 0.001) and 0.756 (95% CI: 0.650, 0.863, *p* < 0.001), respectively. The AUCs of FLIS at 5, 10 and 15 min after enhancement for predicting ICG-R_15_ greater than 40% were 0.728 (95% CI: 0.578, 0.878, *p* = 0.006), 0.755 (95% CI: 0.602, 0.908, *p* = 0.002) and 0.741 (95% CI: 0.579, 0.904, *p* = 0.004), respectively. The AUCs of FLIS at 5, 10 and 15 min after enhancement for predicting ALBI grade 1 were 0.734 (95% CI: 0.641, 0.827, *p* < 0.001), 0.761 (95% CI: 0.673, 0.848, *p* < 0.001) and 0.691 (95% CI: 0.594, 0.788, *p* = 0.001), respectively. The AUCs of FLIS at 5, 10, and 15 min after enhancement for predicting CP A cirrhosis were 0.806 (95% CI: 0.710, 0.902, *p* < 0.001), 0.821 (95% CI: 0.727, 0.915, *p* < 0.001) and 0.829 (95% CI: 0.727, 0.930, *p* < 0.001), respectively. The AUCs of FLIS at 5, 10, and 15 min after enhancement for predicting MELD score of 10 or less were 0.837 (95% CI: 0.757, 0.916, *p* < 0.001), 0.877 (95% CI: 0.800, 0.954, *p* < 0.001) and 0.837 (95% CI: 0.729, 0.944, *p* < 0.001), respectively (Figure 4). No significant difference was found in the area under the ROC curve of FLIS at 5, 10, and 15 min (*p* > 0.05).

**TABLE 7:** Diagnostic performance of FLIS at different time points for the prediction of ICG-R_15_, ALBI grade, CP class and MELD score. AUC, area under curve; CI, confidence interval; SE, standard error; PPV, positive predictive value; NPV, negative predictive value.

Variable	AUC (95%CI)	SE	Cut-off values	Sensitivity (%)	Specificity (%)	Accuracy (%)	PPV (%)	NPV (%)
Predict for ICG-R_15_ ≤ 10%								
FLIS (5 min)	0.838 (0.763, 0.913)	0.038	2.5	79.6	78.7	79.1	82.8	75.0
FLIS (10 min)	0.802 (0.720, 0.884)	0.042	5.5	75.5	75.4	75.5	79.3	71.2
FLIS (15 min)	0.723 (0.629, 0.817)	0.048	5.5	95.9	47.5	69.1	59.5	93.5
Predict for ICG-R_15_ > 20%								
FLIS (5 min)	0.802 (0.714, 0.890)	0.045	1.5	59.5	86.3	77.3	68.8	80.8
FLIS (10 min)	0.828 (0.742, 0.913)	0.044	5.5	86.5	64.4	71.8	55.2	90.4
FLIS (15 min)	0.764 (0.661, 0.868)	0.053	5.5	62.2	89.0	80.0	74.2	82.3
Predict for ICG-R_15_ > 40%								
FLIS (5 min)	0.728 (0.578, 0.878)	0.077	2.5	85.7	52.1	56.4	20.7	96.2
FLIS (10 min)	0.755 (0.602, 0.908)	0.078	4.5	78.6	70.8	71.8	28.2	95.8
FLIS (15 min)	0.741 (0.579, 0.904)	0.083	5.5	64.3	77.1	75.5	29.0	93.7
Predict for ALBI grade 1								
FLIS (5 min)	0.734 (0.641, 0.827)	0.048	2.5	69.0	66.2	67.3	77.6	55.8
FLIS (10 min)	0.761 (0.673, 0.848)	0.045	5.5	73.8	69.1	70.9	81.0	59.6
FLIS (15 min)	0.691 (0.594, 0.788)	0.049	5.5	95.2	42.6	62.7	50.6	93.5
Predict for CP A cirrhosis								
FLIS (5 min)	0.806 (0.710, 0.902)	0.049	1.5	85.0	66.7	80.0	62.5	87.2
FLIS (10 min)	0.821 (0.727, 0.915)	0.048	4.5	78.8	73.3	77.3	56.4	88.7
FLIS (15 min)	0.829 (0.727, 0.930)	0.052	5.5	88.8	73.3	84.5	71.0	89.9
Predict for MELD score ≤10								
FLIS (5 min)	0.837 (0.757, 0.916)	0.041	2.5	58.6	95.7	66.4	37.9	98.1
FLIS (10 min)	0.877 (0.800, 0.954)	0.039	4.5	78.2	87.0	80.0	51.3	95.8
FLIS (15 min)	0.837 (0.729, 0.944)	0.055	5.5	85.1	78.3	83.6	58.1	93.7

## 4 Discussion

The research pointed out that viral hepatitis serves as the most prevalent CLD or LC reason within our hospital, which takes the share of over 90% of all CLD and LC patients. It is also found that most viral hepatitis LC was brought about by HBV (which was almost 91% of all of the viral hepatitis LC cases). In this research, the data indicated that the CLD or LC incidence in males is higher than that in females, which were 93 males vs. 17 females with the ratio being 5.5:1, fitting the results of Wang et al. (2014). The gender difference is likely to be caused by that HBV infection (with a ratio of male to female of 6.7:1) has a higher prevalence within CLD or LC patients. From the perspective of different gender etiology, there is a higher prevalence of viral hepatitis, mixed etiology and alcohol in men, but autoimmune disease, metabolic disease and cryptogenic cirrhosis in women (Wang et al., 2014; Gu et al., 2018; Xu et al., 2022).

It is well-known that during the arterial and portal venous stages, Gd-EOB-DTPA can disperse into intra- and extra-vascular compartments; during the transitional and hepatobiliary stages, it can be absorbed actively by hepatocytes. For patients with normal liver and kidney function, about 50% of the injected dose is excreted through the kidney and the remaining 50% is excreted through the hepatobiliary system. Evidence reveals that during active transport, Gd-EOB-DTPA absorption takes place through the OATP1 B1 and B3 on the sinusoidal membrane of hepatocytes. In addition, sinusoidal backflow and excretion into the biliary system happen through multi-drug resistance protein transporters (MRP2 and 3, respectively) (Reimer et al., 2004; Van Beers et al., 2012). The relative expression level of transmembrane transporters is the principal determinant of hepatocellular accumulation of Gd-EOB-DTPA molecules, which can lead to biliary contrast excretion and liver parenchymal enhancement in HBP. Therefore, biliary contrast excretion and liver parenchymal enhancement are decreased as the transport and uptake mechanism is impaired and the number of hepatocytes is reduced (Motosugi et al., 2009a; Takao et al., 2011; Kim et al., 2013; Choi et al., 2016). Additionally, the deterioration of Gd-EOB-DTPA absorption results in a prolongation of Gd-EOB-DTPA plasma half-life, which induces portal vein hyperintensity in HBP (Lee et al., 2012; Zhang et al., 2018; Yang et al., 2020). Given the Gd-EOB-DTPA mechanism, biliary contrast excretion, liver parenchymal enhancement, and portal vein sign can be interpreted as appropriate parameters for assessing liver function. In this study, EnQS, ExQS and PVsQS demonstrated a weak to moderate negative correlation with ICG-R_15_, ALBI score, CP score and MELD score except for ExQS at 5 min after enhancement, which was in line with those reported in the literature. Moreover, intra- and inter-observer ICCs were >0.90 for FLIS at 5, 10, and 15 min, which was also consistent with previous studies (Bastati et al., 2016; Bastati et al., 2020; Lee et al., 2021; Aslan et al., 2022; Luo et al., 2022).

Previous studies have shown that a delay of 10 min is sufficient for diagnosis in patients without a history of CLD; for patients with mild liver dysfunction, a 15-min delay is enough to diagnose; and for patients with moderate or severe liver dysfunction, > 5 min delay is not effective (Motosugi et al., 2009b; van Kessel et al., 2012; Esterson et al., 2015; Liang et al., 2016). Takatsu et al. (2021) reported that the higher the quantitative liver-spleen contrast ratio (Q-LSC) of delay images at 3 min, the higher the Q-LSC of delay images at 10 and 15 min. Based on the Q-LSC, transitional phase delay images at 3 min on liver MRI can be used to predict the contrast enhancement effect of HBP images. The findings of this study were consistent with those of Takats et al. To be specific, the FLIS and the three FLIS parameters mostly increased over time, and the FLIS of some patients with normal liver function or mild liver dysfunction reached 6 points on the 10-min delay image.

Results indicated no correlation between ExQS at 5 min after enhancement and CP score (*r* = −0.186, *p* > 0.05) and MELD score (*r* = −0.164, *p* > 0.05), and the remaining FLIS parameters at a delay of 5, 10, and 15 min demonstrated a weak to strong correlation with liver function classification. In this research, approximately 80% of ExQS were 0 at 5 min after contrast injection, and 5 min was speculated not to be sufficient for contrast to be excreted into the biliary tract. FLIS had a moderate negative correlation with liver function classification at each time point (−0.587, −0.580, and −0.547 for ICG-R_15_; −0.515, −0.564, and −0.538 for ALBI score; −0.574, −0.622, and −0.641 for CP score; −0428, −0.555, and −0.507 for MELD score; 5-, 10-, and 15-min delay, respectively) (*p* < 0.05). In previous work, FLIS and its three parameters showed a strong correlation with CP score (*r* = −0.80) and ALBI grade (*r* = [−0.976] to [−0.951]) (Lee et al., 2021; Aslan et al., 2022). However, Luo et al. (2022) reported that FLIS was weakly correlated only with ICG-R_15_, ALBI score, and MELD score (*r* = [−0.268] to [−0.219]). This discrepancy is likely to be due to the differences in the scanning time and the study population. In addition, studies also pointed out that there was no significant correlation between ExQS at 5- and 15-min delay and INR and PT (*r* = 0.096 to 0.172, *p* > 0.05), as well as between FLIS and its three parameters at each time point and creatinine (*r* = 0.018 to 0.156, *p* > 0.05), while the remaining image parameters were weakly to moderately correlated with other clinical parameters, at different time point. This conformed to the findings of Bonatti et al. (2021) observing that the degree of liver enhancement during HBP does not significantly change in patients with moderately to severely impaired renal function. Bastati et al. (2016) indicated that the three parameters showed equal weight for transplant-free survival. However, the correlation coefficients of the three parameters showed unequal weight and ExQS showed a lower correlation than EnQS and PVsQS at each time point in this study. Therefore, the present research should focus on the significant correlation with the clinical scoring system that is used for assessing the level of liver function for three FLIS parameters, rather than equal weight.

FLIS showed moderate discriminatory ability between different liver function levels. The AUCs of FLIS at 5, 10 and 15 min after enhancement for predicting ICG-R_15_ of 10% or less were 0.838, 0.802 and 0.723, respectively (*p* < 0.001); those for predicting ICG-R_15_ of greater than 20% were 0.793, 0.824, and 0.756, respectively (*p* < 0.001); those for predicting ICG-R_15_ of greater than 40% were 0.728, 0.755 and 0.741, respectively (*p* < 0.01); those for predicting ALBI grade were 0.734, 0.761, and 0.691, respectively (*p* ≤ 0.001); those for predicting CP A cirrhosis were 0.806, 0.821, and 0.829, respectively (*p* < 0.001); those for predicting MELD score of 10 or less were 0.837, 0.877, and 0.837, respectively (*p* < 0.001). Previous studies have shown that patients have a good liver function and bisector ectomy can be done if ICG-R_15_ is between 0 and 10%; it is a contraindication for major hepatectomy and right sided section ectomy or left sided hepatectomy can be done if ICG-R_15_ is between 10 and 19%; it indicates the poor liver compensatory function, which is a contraindication for liver resection, and enucleation can be done if ICG-R_15_ is greater than 40% (Imamura et al., 2005; Sharma., 2022). FLIS provided acceptable diagnostic performance for detecting patients with ICG-R_15_ of greater than 20% or 40%, which is crucial for treatment planning in hepatocellular carcinoma (HCC) patients and prevention of post-hepatectomy hepatic insufficiency. Through comparing the FLIS and CP scores, Lee et al. (2021) found that AUC, specificity and sensitivity were 0.938%, 94.4%, and 83.7%, respectively, when predicted CLD and CP A cirrhosis by FLIS≥5, and that AUC, specificity and sensitivity were 0.896, 92.9%, and 81.8%, respectively, when predicted CP B cirrhosis between CP B and C cirrhosis by FLIS≥3. In the same way, Aslan et al. (2022) compared FLIS and ALBI scores to find that AUC, specificity and sensitivity were 0.882–0.917, 82.6%–87%, and 83.7%–95.4%, respectively, when predicted ALBI grade 1 by FLIS≥5, and that AUC, specificity and sensitivity were 0.974–0.994, 95.2%–96%, and 100%, respectively, when distinguished ALBI grade 3 from other grades by FLIS≤3. These findings revealed that FLIS may be used clinically to non-invasively assess the liver function reserve. In addition, the area under the ROC curve of FLIS at 5, 10, and 15 min (*p* > 0.05) respectively had no statistical difference. This means similar FLIS diagnostic efficiency at those time points. As a result, we believe that the hepatobiliary period’s observation time can be reduced by using the FLIS to assess liver function.

There are several limitations to this study. Firstly, selection bias may occur due to its retrospective design, but it is less likely because Gd-EOB-DTPA-enhanced hepatic MRI is a routine examination of patients with CLD or focal liver masses or nodules at our institution. Secondly, MRI images of transitional and hepatobiliary phases obtained after 5, 10, and 15 min of contrast were not compared with standard hepatobiliary phase images at 20 min, and thus it is uncertain whether the same purpose could be achieved by shortening the MRI examination time. Thirdly, whether the obtained results apply to uninfected patients remains to be confirmed as more than 90% of patients were infected with hepatitis B or C virus. Fourthly, nearly half of patients lack histologic evidence regarding the etiologic cause of CLD or LC, which reflects the reality of the clinical routine. Fifthly, the number of cases with end-stage liver disease is relatively rare, that is, such patients have less chance of receiving Gd-EOB-DTPA-enhanced MRI than other patients as they are generally not targets for therapeutic intervention. Finally, the sample size is relatively small without external validation. In the future, large data and multi-center clinical studies are required to further verify the findings of this study.

Despite the above-mentioned limitations, this study is the first to compare FLIS at 5, 10, and 15 min after enhancement with the classification system for evaluating liver function. This will be a research pioneer on this topic.

## Conclusion

There is a moderate negative correlation of FLIS derived from Gd-EOB-DTPA-enhanced MRI of delay images at 5, 10, and 15 min with classification systems of hepatic function. It may be used clinically to non-invasively assess the liver function reserve and appropriately classify patients with CLD or LC based on ICG-R_15_, ALBI grade, MELD score, and CP classification. In addition, the observation time of the hepatobiliary period can be reduced by using FLIS to assess liver function.

## Data Availability

The original contributions presented in the study are included in the article/Supplementary Material, further inquiries can be directed to the corresponding author.

## Abbreviations

ALBI, albumin-bilirubin; ROC, receiver operating characteristic curve; AUC, area under the curve; CLD, chronic liver disease; CP, Child-Pugh; CI, confidence intervals; EnQS, enhancement quality score; ExQS, excretion quality score; PVsQS, portal vein sign quality score; FLIS, functional liver imaging score; Gd-EOB-DTPA, Gadolinium-ethoxy benzyl-diethylene triamine pentaacetic acid; HBP, hepatobiliary phase; HCC, hepatocellular carcinoma; ICG-R_15_, indocyanine green 15-min retention rate; ICC, intraclass correlation coefficient; IQR, interquartile ranges; INR, international normalized ratio; LC, liver cirrhosis; MRI, magnetic resonance imaging; MRP, multi-drug resistance protein transporter; MELD, model for end-stage liver disease; OATP, organic anion transport polypeptide; PT, prothrombin time; PACS, picture archiving and communication system; Q-LSC, quantitative liver-spleen contrast ratio; SD, standard deviation; SPAIR, spectral attenuated inversion recovery.
